# The Book World of Medicine and Science

**Published:** 1901-06-08

**Authors:** 


### The Book World of Medicine and Science.

Outlines for Dissectors. By G. G. Parsons, F.R.C.S (London: John Bale, Sons and Danielsson. In four parts. Price 1s. 6d. each net.)

Skill in drawing is an accomplishment which is of much service in many a walk in life. To few, however, is it of such constant utility as to the student of medicine, especially when he is engaged in working at anatomy. Anatomy may be learned by a mere exercise of word memory, and no doubt is so learned by some. But for practical purposes anatomy is of far more use when it is remembered in the form of a series of pictures, in which every detail stands out before the mind's eye, as it did in the dissected subject; and nothing so greatly helps one in obtaining this sort of knowledge of anatomy as sketching from the "part." But some men cannot draw, and for them these "outlines" will be of the greatest service—outlines of the various dissections plainly drawn on a large scale, so that they can be easily filled in with coloured chalks. To all intents and purposes the student who uses these outlines as they are intended to be used, for making actual sketches from the dissected parts, will be in as good a position, so far as driving the pictures into his mind is concerned, as his more artistic companions. All experience goes in favour of map-drawing in the teaching of geography, and elementary anatomy, as learned in the first instance, and as generally dealt with by examiners, is but the geography of the body. We cordially recommend these aids to study.

Diseases of the Heart: A Clinical Text-book for the use of Students and Practitioners of Medicine. By Edmund Henry Colbeck, B.A., M.D. (Cantab), M.R.C.P. London. (London: Methuen and Co. 1901. Price 12s. Pp. 336. Illustrations 43.)

At the present day it is difficult to discover a genuine hiatus in the serried ranks of medical literature. Dr. Colbeck is therefore to be congratulated on having produced a text-book on the heart which students with little or no clinical experience have long wanted, and have had to do without. The author has adopted a system from which he has never allowed himself to depart. Each section is complete in itself and modelled on a uniform plan, and although this method may have entailed some reiteration and may have made the book somewhat dull reading, nevertheless, from the student's point of view, it is exactly the thing which is wanted. We only regret that no such text-book was in existence when we ourselves were struggling with the difficulties of mitral stenosis and aortic regurgitation. Being a work for students, controversial subjects are wisely omitted, and the author has seldom given rein to his imagination, or to the expression of original views or theories. We believe, however, that his views on the relationship of mitral stenosis and mitral insufficiency are new—at least we cannot recollect having seen a similar expression of opinion elsewhere. This is what is said as regards this subject on page 181. "A more likely cause of irritation of the valvular segments is the more or less constant vibration of these structures (mitral flaps) that is maintained by the to-and-fro flow of blood, which accompanies the mitral insufficiency so commonly associated with anæmia. Moreover, it is to be expected that an overgrowth of fibrous tissue in the valvular curtains and basal ring would be more readily excited in young anæmic subjects than in adults by irritation of this kind." The theories which he submits as an explanation of the pathogenesis of angina pectoris, and the combined use of various drugs in the treatment of cardiac failure, if not actually new are at least originally dealt with. In the section which is devoted to diseases of the myocardium, Dr. Mitchell Bruce's lucid explanation of the production of dilatation and hypertrophy as well as his nomenclature has been adopted by the author. This simplification of what is often the "pons asinorum" of medical students, will be much appreciated by those who try to master the subject. An excellent feature in the chapters which deal with valvular disease, is the attention which is devoted to questions of prognosis. Students, and indeed many practitioners, are frequently unable to discriminate between the prognostic significance of the various features of a case of morbus cordis. The murmur itself generally takes a position of undue importance. The general effects upon the circulation, and the contributory or secondary features, etiological and pathological, seldom receive the attention to which they are justly entitled. To take an all-round view of every case of heart disease is one of the points on which Dr. Colbeck chiefly insists. And to enable the student to gauge accurately the degree of stenosis or insufficiency at any particular orifice the methods long ago suggested by Sir William Broadbent have been systematised and extended. The reader will be thus enabled, if he follows the directions supplied in these pages, to form a very fair estimate of the probable course of independent or combined lesions. This systematic arrangement should have a sphere of usefulness quite outside the limits of general practice. The work is well illustrated, and contains several original and ingenious schematic diagrams.

